# In Vitro Antioxidant Activity and In Vivo Anti-Fatigue Effect of Sea Horse (Hippocampus) Peptides

**DOI:** 10.3390/molecules22030482

**Published:** 2017-03-18

**Authors:** Zebin Guo, Duanquan Lin, Juanjuan Guo, Yi Zhang, Baodong Zheng

**Affiliations:** 1 College of Food Science, Fujian Agriculture and Forestry University, Fuzhou 350000, Fujian, China; gzb8607@163.com (Z.G.); duanquan11@gmail.com (D.L.); gjjfst15@163.com (J.G.); zyifst@163.com (Y.Z.); 2 Engineering Research Center of Marine Living Resources Integrated Processing and Safety Risk Assessment, Fuzhou 350002, Fujian, China

**Keywords:** peptide, *Hippocampus*, antioxidant activity, anti-fatigue effect

## Abstract

This study investigated changes the in vitro antioxidant activity of *Hippocampus* polypeptides during enzymatic hydrolysis, including the effects of enzyme species, enzyme concentration, material–liquid ratio, hydrolysis time, pH, and temperature of the reaction system. Its in vivo anti-fatigue activity was also studied. *Hippocampus* peptide prepared by papain digestion exhibited the highest 1,1-diphenyl-2-picryl-hydrazyl free radical scavenging rate (71.89% ± 1.50%) and strong hydroxyl radical scavenging rate (75.53% ± 0.98%), compared to those prepared by five other commonly used enzymes (i.e., trypsin, neutral protease, compound protease, flavorzyme, and alkaline protease). Additionally, maximum antioxidant activity of *Hippocampus* polypeptide prepared by papain digestion was reached after hydrolysis for 40 min at pH 6.0 and 60 °C of the reaction system by using 2000 U/g enzyme and a material–liquid ratio of 1:15. Moreover, compared with the control group, *Hippocampus* peptide prolonged the swimming time by 33%–40%, stabilized the blood glucose concentration, increased liver glycogen levels, and decreased blood lactate levels and blood urea nitrogen levels in mice (*p* < 0.01). In conclusion, these results indicated that *Hippocampus* polypeptide prepared by papain digestion under optimal conditions exhibited high degrees of antioxidant and anti-fatigue activity.

## 1. Introduction

Fatigue always occurs upon reaching a certain peak of mental or physical status [1], which not only marks a temporary decrease in work ability, but may also be a precursor to several diseases [2]. With the rapid development of modern society, fatigue and stress are becoming increasingly common health issues [3,4,5]. Therefore, the development of effective anti-fatigue products is very important. Anti-fatigue peptides are currently popular research topics. Bioactive peptides are derived from three main sources: (1) natural or endogenous bioactive peptides, (2) exogenous bioactive peptides prepared through protein hydrolysis, and (3) synthetic peptides made by some chemical methods (e.g., solution- or liquid-phase peptide synthesis and solid-phase peptide synthesis) [6]. Due to low yields, the poor stability and potential toxicity of endogenous bioactive peptides resulted in high costs and increased challenges for industrial-level production. Thus, enzymatic hydrolysis has become a widely used method for bioactive peptide generation. Bioactive peptides have a range of physiological and biochemical functions, including antimicrobial, anti-fatigue, anti-tumor, antioxidant, antihypertensive, and immunomodulatory activities [7,8,9,10,11]. They have been shown to exert anti-fatigue effects by providing energy, promoting the removal of metabolic waste and excess free radicals, and enhancing antioxidant capacity [12,13].

*Hippocampus* belongs to the *Syngnathidae* family and provides a source of traditional Chinese medicine materials. *Hippocampus* is rich in proteins and essential amino acids. It is a high-quality material for preparation of proteins and related products. Moreover, previous studies have reported that a high ratio of heterocyclic or aromatic (i.e., His, Pro, Tyr, and Phe) and acidic (i.e., Glu and Asp) amino acids accounted for 16.14% and 20.09% of the total amino groups in *Hippocampus*, respectively. *Hippocampus* hydrolysates could quench free radicals and chelate metals, supporting their utility as antioxidants and antioxidant agents [14,15]. Recent studies have shown that amino acids, trace elements, unsaturated fatty acids, and other functional components in *Hippocampus* contributed to its hormone-like, hematopoiesis, anti-aging, anti-fatigue, and Ca^2+^-blocking functions [16]. However, there is further study about the use of *Hippocampus* polypeptide for anti-fatigue treatment. In this study, the optimal enzymatic hydrolysis conditions for preparation of *Hippocampus* polypeptide and its anti-fatigue activity were investigated.

## 2. Results and Discussion

### 2.1. Preparation of Hippocampal Polypeptides

**Figure 1:**
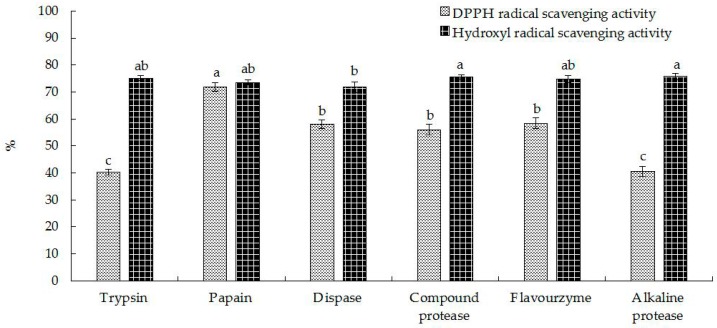
Antioxidant activities of *Hippocampus* hydrolysates prepared by trypsin, papain, neutral protease, complex protease, flavor protease, and alkaline protease (*p* < 0.05).

Proteases exhibit cleavage site specificity, leading to hydrolysates with distinct physicochemical, biological, and functional properties [17,18,19]. Thus, the selection of an appropriate protease was a key for the preparation of bioactive peptides with high-activity. As shown in Figure 1, this study was focused on the effects of papain, trypsin, neutral protease, complex protease, flavor protease, and alkaline protease on the antioxidant activity of *Hippocampus* proteins. There were no significant differences in peptide yield, which meant these enzymes had similar cleavage effects on *Hippocampus* proteins. However, the peptide generated by papain digestion had the highest DPPH free radical scavenging (71.89% ± 1.50%) and hydroxyl radical scavenging (75.53% ± 0.98%) rates compared to those prepared by the five other proteases. Consequently, papain was selected as the proteolytic enzyme for the preparation of *Hippocampus* polypeptide.

Excessive accumulation of free radicals leads to lipid peroxidation in the plasma membrane and destruction of the natural structures of intracellular proteins and nucleic acids, thus decreasing the permeability of the mitochondrial inner membrane and causing oxidative fatigue. Active peptides can quench free radicals or block the growth of free radical chains in lipids to protect cells from oxidative damage, which results in increasing antioxidant capacity and improving anti-fatigue ability. Ding et al. [12] found that jellyfish collagen hydrolysate improved anti-fatigue ability and increased superoxide dismutase and glutathione peroxidase activities in mice that were compared with a senile model group. The effects of pH and temperature of the reaction system, time of hydrolysis, enzyme dose, and solid–liquid ratio on DPPH free radical scavenging, hydroxyl radical scavenging, degree of hydrolysis, and peptide yield were presented in Table 1. The hydroxyl radical scavenging rates of hydrolysate increased slightly with increasing pH values of the reaction system. However, the DPPH radical scavenging rate of the hydrolysate was significantly decreased at pH 6.0. The result of the degree of hydrolysis showed that substrate hydrolysis was insufficient at pH > 6.0, precluding full release of polypeptides with strong DPPH scavenging activity. Additionally, the temperature of the reaction system had no significant impact on the DPPH scavenging rate of hydrolysate. However, the hydroxyl scavenging activity of hydrolysate showed a bell-shape trend (an initial increase followed by a decrease) with increasing temperature, and reached the maximum hydroxyl scavenging rate at 60 °C. One possible explanation for this result is that the low temperature (<60 °C) leaded to insufficient hydrolysis and precludes full release of target peptides with strong antioxidant activity, while the high temperatures (>60 °C) could result in excessive hydrolysis of target peptides to amino acids, and thus reducing the antioxidant activity. With increasing time of hydrolysis and enzyme dosage, DPPH free radical and hydroxyl free radical scavenging rates and the peptide yield decreased, while the degree of hydrolysis increased significantly; this indicated that longer reaction times and higher enzyme doses resulted in excessive substrate hydrolysis, suppressing the antioxidant activity of polypeptides. The increase of the material–liquid ratio showed no significant differences in the hydroxyl scavenging rate. However, when the material–liquid ratio exceeded 1:10, the DPPH radical scavenging rate of hydrolysate decreased sharply. Moreover, peptide yield remained stable with increasing degrees of hydrolysis, indicating that enzymatic efficiency was enhanced with increasing material–liquid ratio. In this case, the substrate was potentially hydrolyzed excessively, which lead to a decrease DPPH free radical scavenging rate. These findings suggested that polypeptides with specific molecular weight distributions, exhibiting the highest biological activity, and either excessive or insufficient hydrolysis, could potentially result in lower bioactivity. Therefore, during the hydrolyzation, reaction conditions and degree of hydrolysis should be controlled to obtain peptides within the target molecular weight range. Considering the cost, efficiency, and subsequent preparation processes, the optimal hydrolysis conditions for producing *Hippocampus* polypeptides with high antioxidant activity were as follows: pH 6.0, 60 °C, reaction time of 40 min, enzyme dosage of 2000 U/g, and material–liquid ratio of 1:15.

### 2.2. Anti-Fatigue Activity of Hippocampus Peptide

**Figure 2:**
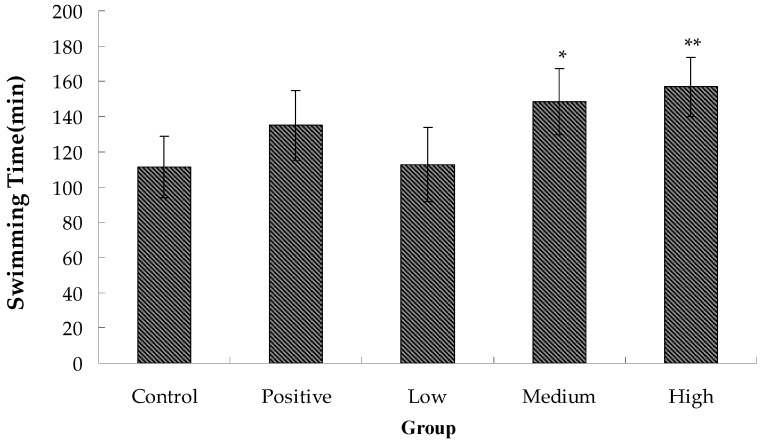
Effect of *Hippocampus* peptides on the swimming time of mice as compared to the control group (* *p* < 0.05; ** *p* < 0.01).

During prolonged exercise, fatigue occurs as a result of increased oxygen, blood glucose, and protein consumption by muscles. Moreover, exercise triggers hypoxia and the generation of excessive free radicals, which affects protease dehydrogenation and induces changes in intracellular osmotic pressure, inhibition of cell respiration and decreased adenosine triphosphate (ATP) synthesis. The decrease of exercise endurance is the most direct and objective indicator of fatigue. As shown in Figure 2, the swimming time of the negative control group was 111.33 ± 17.35 min, which was lower than that of the positive control and experimental groups. Furthermore, swimming time of mice was prolonged with increasing levels of *Hippocampus* peptide. The medium- and high-dose groups also differed significantly from the blank group, with increases by 33.39% and 40.87% in swimming time, reaching 148.50 ± 18.72 min and 156.83 ± 16.70 min, respectively. These results indicated that while both glutathione and *Hippocampus* peptide enhanced exhaustive swimming time and exercise tolerance in mice, *Hippocampus* peptide showed a more significant effect.

Before feeling fatigue, a variety of intrinsic physiological and biochemical reactions occur in the body. In this paper, the changes in contents of blood glucose, blood lactic acid, serum urea nitrogen, and hepatic glycogen in mice before and after exercise were measured to determine the anti-fatigue activity of *Hippocampus* peptide.

**Figure 3:**
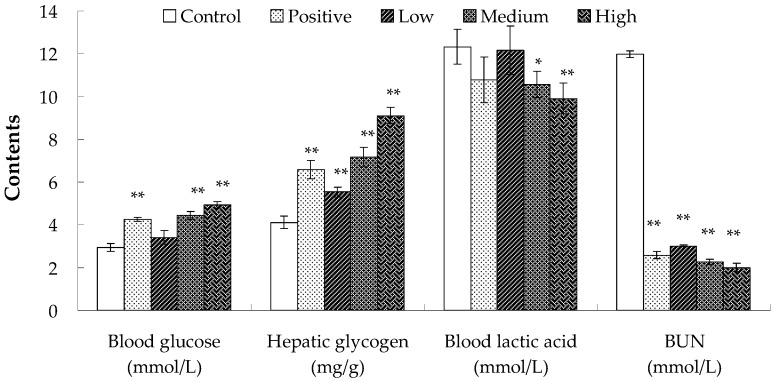
Effect of *Hippocampus* peptides on blood glucose, hepatic glycogen, blood lactic acid, and serum urea nitrogen of mice post-exercise, as compared to the control group (* *p* < 0.05; ** *p* < 0.01).

Blood glucose is one of the main sources of energy supply for the central nervous system and is the only source of energy supply for red blood cells. Therefore, stability of blood glucose level is critical for the functioning of organs [20]. Prolonged exhaustive exercise leads to the consumption of large amounts of carbohydrates, potentially resulting in hypoglycemia without supplementation of exogenous sugar. A decline in blood glucose directly affects the energy supply to brain cells, resulting in reduced activity of pallium and physical fatigue. The importance of hepatic glycogen is that it could be broken down into glucose to maintain blood glucose level when in hunger or exercising [21]. Hepatic glycogen storage and its decomposition rate can directly affect exercise capacity and duration [22]. Therefore, contents of glucose and hepatic glycogen can reflect the level of fatigue [23]. Blood glucose levels in the blank and low-dose groups did not have significant difference and were lower than the normal range (3.7–6.9 mmol/L) (Figure 3). The blood glucose levels in the positive control, medium-dose, and high-dose groups were all within the normal range, which had significant differences compared to that in the blank group. Furthermore, hepatic glycogen levels increased with increasing levels of *Hippocampus* peptide. These findings confirmed those results of swimming experiments. Thus, glutathione and *Hippocampus* peptide could improve utilization of hepatic glycogen or increase hepatic glycogen storage to stabilize blood glucose concentration in mice, and enhance their exercise tolerance.

High-intensity or extended exercise increases oxygen consumption in muscles, resulting in hypoxia, acceleration of glycolysis, and generation of large amounts of acidic substances such as lactic acid and pyruvate. These changes decrease the pH value and contractility of muscle, alter homeostasis, and reduce exercise capacity [24,25,26]. Therefore, accumulation of lactic acid is an important contributory factor to physical fatigue [23]. As shown in Figure 3, the lactic acid content of mice in the blank group was 12.32 ± 0.81 mmol/L, which was higher than that in the positive control and experimental groups. In addition, the lactic acid levels in the experimental groups decreased with increasing levels of *Hippocampus* peptide. The difference between the positive control and blank groups was not significant. The lactic acid level in the positive control group was decreased by 12.50%, and the level in the low-dose group was only slightly lower than that in the blank group. The differences between the medium- or high-dose and blank groups were significant, with blood lactate levels decreased by 14.12% and 19.56%, respectively, reaching only 10.58 ± 0.61 mmol/L and 9.91 ± 0.72 mmol/L. These results indicated that while both glutathione and *Hippocampus* peptide improved exercise tolerance by reducing accumulation of lactic acid, *Hippocampus* peptide was more effective; this is possibly because *Hippocampus* peptide has high antioxidant activity and can inhibit generation of reactive oxygen species. Additionally, *Hippocampus* peptide was able to clear free radicals produced during excessive exercise, and reduced the lactate production rate, thereby enhancing exercise tolerance.

Adenylate catabolism is elevated during prolonged exercise, and when glycogen storage is insufficient, protein decomposition occurs to produce energy. The metabolism of protein and amino acid produces NH_3_ and CO_2_, leading to the synthesis of urea in liver, which is expelled through blood circulation and the kidneys [26,27]. Under normal physiological conditions, the formation and excretion of urea remain in equilibrium. However, after prolonged exercise, if protein oxidation increases to meet energy demands, urea (including blood urea) levels rise significantly. The content of blood urea nitrogen could show the level of blood urea, which is significantly negatively correlated with exercise tolerance [28]. As shown in Figure 3, the levels of blood urea nitrogen in the blank, positive control, low-, medium-, and high-dose groups were 11.97 ± 0.15 mmol/L, 2.56 ± 0.17 mmol/L, 3.01 ± 0.05 mmol/L, 2.26 ± 0.13 mmol/L, and 2.00 ± 0.20 mmol/L, respectively, which clearly show a significant decrease in blood urea nitrogen in the positive control and experimental groups. The results indicated that a large amount of urea nitrogen was generated in the blank group due to the increased protein oxidation after exercise. However, the levels of blood urea nitrogen in the positive control and experimental groups remained low, which was possibly due to sufficient energy supplies from the oxidative dehydrogenation of glutathione and *Hippocampus* peptide, thereby precluding the decomposition protein [29].

## 3. Materials and Methods

### 3.1. Materials

Dried *Hippocampus* was provided by Fujian East Asia Fisheries Co., Ltd. (Dongshan, China), and Institute for Cancer Research (ICR) mice (male, weighing between 18 and 22 g) were purchased from the Experimental Animal Center of Fujian Medical University (Fuzhou, China). Blood glucose, blood lactate, serum urea nitrogen, and liver glycogen test cartridges were obtained from Nanjing Jiancheng Biological Engineering Research Institute (Nanjing, China). All chemical agents used in this paper were of analytical grade.

### 3.2. Methods 

#### 3.2.1. Preparation of Hippocampus Polypeptide

Dried *Hippocampus* was crushed before enzymolysis, and enzymolysis was performed under different conditions (Table 2). Incubation was performed in a water bath with reciprocal shaking, and the hydrolysis reaction was terminated by heating at 100 °C for 15 min. Solutions were cooled to room temperature and centrifuged (Allegra X-30R Centrifuge, Beckman Coulter, Inc., Fullerton, CA, USA) at 3600× *g* for 20 min. The supernatant was collected, and degree of hydrolysis, peptide yield, 1,1-diphenyl-2-picryl-hydrazyl (DPPH) free radical scavenging, and hydroxyl radical scavenging rates were measured.

After establishing the optimal conditions, enzymatic hydrolysis of *Hippocampus* proteins was performed according to a previously described procedure, and the supernatant purified using 10,000 Da and 5000 Da ultrafiltration membranes. The filtrate was lyophilized to obtain *Hippocampus* polypeptide powder with the molecular weight <5000 Da. The polypeptide concentration was estimated to be 83.53% ± 1.53% using the Folin phenol reagent method [30]. *Hippocampus* polypeptide powder was used for the animal experiment to determine its anti-fatigue activity.

#### 3.2.2. Determination of the Degree of Hydrolysis

The degree of hydrolysis was measured using a previously established method with minor modifications [31]. Enzyme solution (2 mL) and distilled water (15 mL) were put in a 50 mL beaker, and the pH value was adjusted to 8.2 using 0.1 M NaOH. Formaldehyde (2 mL, pH 8.2) was added, following by adjusting the pH value to 9.2 using 0.1 M NaOH and recording the amount of NaOH solution (V). Distilled water was used as the blank group to replace enzyme solution, and the amount of NaOH solution (V_0_) was recorded as well. Total free amino nitrogen content of the hydrolysate was calculated as: Ammonia nitrogen content (mg/mL) = 1.4008 × (V − V_0_)/2 (1 mL of 0.1 M NaOH solution = 1.4008 mg of nitrogen). The degree of hydrolysis was measured as: DH (%) = ( A_1_ − A_0_)/A × 100, whereby A_1_ represents the content of free amino nitrogen in the hydrolysate, A_0_ represents the amino nitrogen content in the solution prior to hydrolysis, and A represents the total nitrogen content of *Hippocampus* protein.

#### 3.2.3. Determination of Peptide Yield

Enzymatic solution (2 mL) was transferred to a test tube, and 4 mL of 10% (*w*/*w*) trichloroacetic acid (TCA) solution was added to the test tube. The mixture was shaken and incubated for 0.5 h before centrifugating at 3600× *g* for 10 min to remove protein via TCA precipitation. The above enzymatic solution or water (1 mL) was added to the tube, followed by adding 4 mL Biuret reagent. The tube was shaken and stored at room temperature (20–25 °C for 30 min), followed by colorimetric determination at 540 nm. Another tube containing water was used as a control. The amount of polypeptide was measured using a standard curve of the content of casein phosphopeptide. The experiment was repeated three times. The content of *Hippocampus* protein was estimated to be 72.24% ± 1.08% using the Kjeldahl method [32], and peptide yield was calculated as: Peptide yield (%) = polypeptide in the hydrolysate/total protein content of raw materials.

#### 3.2.4. Measurement of the Hydroxyl Free Radical Scavenging Rate

A previous method for measuring hydroxyl radical scavenging activity was modified [33]. Two milliliters of the hydrolysate was treated with 2 mL of 3 mM FeSO_4_ solution, 2 mL of 6 mM salicylic acid-ethanol solution, and 2 mL of 3 mM peroxide, and the reaction incubated at 37 °C for 30 min. Distilled water was used as the reference solution, and absorbance (Ai) was measured at 510 nm. The absorbance of background (Aj) was measured in solution containing 2 mL distilled water, 2 mL of 3 mM FeSO_4_ solution, 2 mL of 6 mM salicylic acid-ethanol solution, and 2 mL of the hydrolysate after incubating at 37 °C for 30 min. The absorbance of the blank group (Ao) was measured in solution containing 2 mL distilled water, 2 mL of 3 mM FeSO_4_ solution, 2 mL of 6 mM salicylic acid-ethanol solution and 2 mL of 3 mM H_2_O_2_ solution. The scavenging rate for hydroxyl radicals by *Hippocampus* hydrolysate was calculated using the formula:

(1) $$\text{Hydroxyl radical scavenging rate}=\left(1-\frac{Ai-Aj}{Ao}\right)\times 100$$

where Ai represents absorbance of the hydrolysate after adding FeSO_4_ solution, salicylic acid-ethanol solution, and hydrogen peroxide; Aj represents tthe background absorbance; and Ao represents the absorbance of the blank group.

#### 3.2.5. Measurement of the DPPH Scavenging Rate

A previous method for determining DPPH free radical scavenging activity was modified [34]. The hydrolysate (2 mL; diluted 10-fold) was mixed with 2 mL of 1 × 10^−4^ M DPPH-ethanol solution and incubated at room temperature for 30 min in the dark. Absorbance (Ai) was measured at 517 nm. In the blank group (Aj), DPPH solution was replaced with an equal volume of absolute ethanol. In the control group (Ao), the hydrolysate was replaced with an equal volume of absolute ethanol. The DPPH free radical scavenging rate was calculated as follows:

(2) $$\text{DPPH-free-radical-scavenging rate}=\left(1-\frac{Ai-Aj}{Ao}\right)\times 100$$

where Ai represents the absorbance of sample, Aj represents the absorbance of the blank group, and Ao represents the absorbance of control group.

#### 3.2.6. Animal-Experimental Design

ICR mice were divided into five groups, each of 20. In the blank group, mice were administered 10 µL/g of distilled water. Mice in the positive control group were fed 0.5 mg/g of glutathione. Glutathione is an important anti-fatigue peptide, and contains an active thiol group (-SH) that can be easily oxidized and dehydrogenated, thus scavenging free radicals and eliminating peroxidation. Glutathione is also involved in the TCA cycle and glucose metabolism, and can activate the activity of enzyme to produce more energy production and relieve exercise-induced fatigue rapidly. Mice in the other three groups were fed 0.15 mg/g, 0.5 mg/g, or 1.5 mg/g of *Hippocampus* polypeptide, representing low-, medium- and high-dose groups, respectively. Five mice were kept per cage and standard mouse foods were available to them. The frequency of gavage was once per day, and the duration of administration was 4 weeks. All mice were weighed once every week for 4 weeks. The behavioral characteristics of mice were observed daily. After the last gavage, 10 mice in each group were subjected to a swimming exhaustion test, and the other 10 animals were used to determine the changes in physiological and biochemical indexes, including blood glucose, blood lactic acid, serum urea nitrogen, and hepatic glycogen, after a 30 min swimming test.

#### 3.2.7. Swimming Exhaustion Experiment

Mice were allowed to rest for 30 min after the last gavage, and 10 animals per group placed in a swimming pool (water temperature was 25 ± 1 °C; water depth was ≥30 cm). The water was constantly stirred to ensure continual exercise of mice until mice sank for over 10 s and no longer floated, which was regarded as the standard for exhaustion to the point of death. The time from the start point of swimming to death of mice was recorded as the swimming exhaustion time. Mice were rescued from the pool, dried by paper towels, and put in their cages.

#### 3.2.8. Determination of Physiological and Biochemical Indices

Mice were allowed to rest for 30 min after the last gavage, and the other 10 animals in each group placed in a swimming pool (water temperature was 25 ± 1 °C; water depth was ≥30 cm). We constantly agitated the water to ensure continual movement of mice. After swimming for 30 min, mice were removed from the pool and dried with paper towels. Blood samples were taken from the eyeball, and livers were collected via biopsy. Whole blood was centrifuged at 2500× *g* for 15 min to obtain serum, and collected samples were stored at −80 °C. The preservation period was ≤1 month. Contents of blood glucose, blood lactic acid, serum urea nitrogen, and hepatic glycogen were measured according to the manufacturer's instructions.

### 3.3. Statistical Analysis

Results were expressed as means ± standard deviation. Differences between groups were evaluated using analysis of variance and *Tukey* test. *p* values < 0.05 were considered statistically significant.

## 4. Conclusions

Our results showed that peptides with a specific molecular weight distribution, which exhibited the highest bioactivity, and neither excessive nor insufficient hydrolysis, were beneficial for the release of *Hippocampus* peptides within the requisite molecular weight range. Therefore, hydrolytic conditions should be stringently controlled to obtain the maximal amounts of peptide products with target molecular weight distribution. *Hippocampus* peptides prepared via papain digestion exhibited elevated antioxidant and anti-fatigue activities. This is possibly explained by *Hippocampus* peptides stabilizing blood glucose concentration, improving the utilization or storage capacity of hepatic glycogen, reducing accumulation of lactic acid, and providing adequate energy. However, the specific mechanisms and pathways underlying these effects need further study. 

## Figures and Tables

**Table 1:** Effects of pH and temperature of the reaction system, time of hydrolysis, enzyme dose, and solid–liquid ratio on 1,1-diphenyl-2-picryl-hydrazyl (DPPH) free radical scavenging, hydroxyl radical scavenging, degree of hydrolysis, and peptide yield of *Hippocampus* peptides prepared by papain digestion.

Factors/Levels	DPPH Free Radical Scavenging Rate (%)	Hydroxyl Radical Scavenging Rate (%)	Degree of Hydrolysis (%)	Yield of Peptide (%)
pH				
5.0	88.45 ± 1.43 ^a^	72.46 ± 0.44 ^cd^	16.35 ± 0.43 ^ab^	33.12 ± 0.85 ^a^
5.5	87.29 ± 0^.^50 ^a^	71.52 ± 0.71 ^d^	17.48 ± 0.75 ^a^	33.03 ± 0.63 ^a^
6.0	83.46 ± 0.93 ^b^	74.17 ± 0.70 ^c^	17.70 ± 0.50 ^a^	32.55 ± 0.50 ^a^
6.5	70.98 ± 0.58 ^c^	74.34 ± 0.64 ^c^	16.72 ± 0.82 ^ab^	32.96 ± 0.88 ^a^
7.0	56.07 ± 0.70 ^d^	80.68 ± 0.31 ^b^	16.54 ± 0.27 ^ab^	33.42 ± 0.36 ^a^
7.5	42.08 ± 0.90 ^e^	83.30 ± 1.10 ^a^	15.81 ± 0.36 ^b^	33.97 ± 0.64 ^a^
Temperature (°C)				
50	73.76 ± 0.36 ^a^	75.79 ± 1.19 ^ab^	16.21 ± 0.42 ^c^	34.06 ± 0.23 ^a^
55	74.49 ± 0.24 ^a^	76.22 ± 0.52 ^a^	16.65 ± 0.27 ^bc^	33.81 ± 0.41 ^ab^
60	74.49 ± 0.48 ^a^	76.96 ± 0.26 ^a^	17.27 ± 0.61 ^abc^	32.96 ± 0.28 ^bc^
65	74.61 ± 0.85 ^a^	74.24 ± 0.42 ^bc^	18.14 ± 0.42 ^a^	32.87 ± 0.37 ^c^
70	74.72 ± 0.26 ^a^	73.67 ± 0.49 ^c^	17.70 ± 0.21 ^ab^	33.01 ± 0.31 ^bc^
75	74.49 ± 1.13 ^a^	74.40 ± 0.46 ^bc^	16.97 ± 0.51 ^abc^	32.60 ± 0.39 ^c^
Time of hydrolysis (min)				
20	69.69 ± 1.04 ^a^	73.87 ± 0.29 ^b^	13.69 ± 0.28 ^e^	34.88 ± 0.66 ^a^
40	71.48 ± 0.73 ^a^	76.53 ± 0.88 ^a^	15.08 ± 0.41 ^d^	34.61 ± 0.28 ^a^
60	70.28 ± 0.84 ^a^	77.18 ± 0.84 ^a^	16.21 ± 0.65 ^cd^	33.69 ± 0.58 ^a^
80	69.36 ± 0.55 ^a^	73.20 ± 1.01 ^b^	17.38 ± 0.25 ^bc^	31.98 ± 0.74 ^b^
100	65.30 ± 0.89 ^b^	72.18 ± 0.90 ^bc^	18.14 ± 0.14 ^ab^	31.93 ± 0.16 ^b^
120	60.30 ± 0.96 ^c^	70.34 ± 0.31 ^c^	19.13 ± 0.85 ^a^	31.73 ± 0.46 ^b^
Enzyme concentration (U/g)				
1000	73.57 ± 0.34 ^a^	78.43 ± 0.54 ^a^	12.30 ± 0.49 ^d^	33.30 ± 0.47 ^a^
2000	73.38 ± 1.09 ^a^	77.94 ± 0.46 ^a^	14.02 ± 0.35 ^c^	33.10 ± 0.22 ^ab^
3000	72.93 ± 0.28 ^ab^	77.56 ± 0.52 ^a^	14.89 ± 0.57 ^c^	32.07 ± 0.83 ^abc^
4000	71.40 ± 0.64 ^bc^	75.12 ± 0.23 ^b^	16.35 ± 0.12 ^b^	31.84 ± 0.60 ^bcd^
5000	70.96 ± 0.22 ^c^	75.03 ± 0.76 ^b^	16.79 ± 0.35 ^b^	31.61 ± 0.23 ^cd^
6000	68.28 ± 0.58 ^d^	74.87 ± 0.60 ^b^	18.21 ± 0.62 ^a^	30.61 ± 0.42 ^d^
Solid-liquid ratio				
1:5	78.15 ± 0.59 ^a^	70.43 ± 0.27 ^d^	13.54 ± 0.66 ^f^	13.12 ± 0.49 ^e^
1:10	78.21 ± 0.41 ^a^	74.24 ± 0.65 ^c^	15.20 ± 0.32 ^e^	23.86 ± 0.31 ^d^
1:15	74.39 ± 0.56 ^b^	76.51 ± 0.31 ^b^	16.72 ± 0.43 ^d^	32.34 ± 0.33 ^c^
1:20	69.00 ± 0.29 ^c^	76.53 ± 0.46 ^b^	18.22 ± 0.66 ^c^	34.53 ± 0.62 ^b^
1:25	62.12 ± 1.15 ^d^	76.67 ± 0.60 ^b^	20.80 ± 0.37 ^b^	35.84 ± 0.64 ^ab^
1:30	54.10 ± 0.29 ^e^	84.52 ± 0.38 ^a^	23.15 ± 0.68 ^a^	36.29 ± 0.41 ^a^

The results represent the average of triplicates ± standard deviation of three independent assays. Values followed by different small letters in columns indicate significant difference (*p* < 0.05) by Tukey-test.

**Table 2:** Characteristics and suitable reaction conditions of commonly-used proteases.

Protease	Loading Amount (U/g)	pH	Enzymolysis Time (h)	Enzymolysis Temperature (℃)	Material/Solvent
Neutral protease	4000	7.0	2	50	1:15
Flavorzyme	4000	7.0	2	50	1:15
Compound protease	4000	7.0	2	50	1:15
Papain	4000	6.5	2	55	1:15
Trypsin	4000	7.5	2	45	1:15
Alkaline protease	4000	8.0	2	50	1:15
